# The Association Between IL‐8 Gene Polymorphisms and the Risk of Several Types of Cancer, Especially in Gastric Cancer

**DOI:** 10.1002/cnr2.70103

**Published:** 2025-01-17

**Authors:** Bin Xu, Yidan Yan

**Affiliations:** ^1^ Geriatrics Department Affiliated Hospital of Jiangnan University Wuxi China; ^2^ Medical Oncology Affiliated Hospital of Jiangnan University Wuxi China

**Keywords:** biomarker, cancer susceptibility, gastric cancer, IL‐8, polymorphism

## Abstract

**Background:**

Changes in functional genetic polymorphisms may increase or decrease the risk of cancer in patients. Nowadays, the association between polymorphisms in the interleukin‐8 (IL‐8) gene and the susceptibility of cancer risk have been investigated in many studies, however, above relationships remain unclear.

**Aim:**

The current study aims to comprehensively evaluate the association between IL‐8 gene six polymorphisms and the whole cancer risk, especially −251 polymorphism and gastric cancer.

**Methods and Results:**

Six polymorphisms (−251, −353, +678, +1633, +2767, +781) were collected. The expression of serum IL‐8 was calculated by ELISA assay. First, 104 case–control studies were conducted. Second, this research has made significant discoveries regarding the −251, −353 and +781 polymorphisms and the potential associations with cancer risk. Finally, the serum IL‐8 levels in gastric cancer patients with AA/TT genotypes were significantly higher than those with the same genotypes of healthy controls and TT genotypes in gastric cancer patients.

**Conclusion:**

Overall, the investigation has revealed that IL‐8 gene polymorphisms significantly influence vulnerability to cancer development, especially for gastric cancer.

## 1 Introduction

Cancer, a broad range of diseases, can originate in nearly any tissue or organ within the human body. Cancer cells have the capability to disseminate to distant organs, thereby establishing secondary tumor sites [1, 2]. The subsequent phenomenon is referred to as metastasis, which significantly contributes to mortality in cancer patients. A neoplasm, also referred to as a malignant tumor, is a prevalent term used to describe the pathological condition known as cancer. In 2020, a global estimation revealed that almost 19.3 million new cancer instances were diagnosed, with an undesirable mortality rate of 10.0 million cancer patients [2]. Among the diverse array of cancer types, prostate, lung, stomach, colorectal, and liver cancer exhibit the highest prevalence in males. Conversely, breast, thyroid, colorectal, cervical, and lung cancer are the predominant neoplastic conditions commonly encountered in females [3].

According to current scientific literature, a significant proportion, ranging from 30% to 50%, of mortality resulting from malignant neoplastic diseases can be prevented by altering or avoiding pivotal risk factors. Furthermore, the implementation of established prevention strategies that are firmly grounded in empirical evidence may also involve in the reduction of cancer‐related fatalities. Reducing the cancer burden can be achieved by implementing strategies for early cancer detection and effectively managing individuals who develop cancer.

The early detection of cancer plays a dynamic role in signifying the efficacy of treatment interventions, thereby increasing the possibility of survival while minimizing morbidity and the financial burden associated with treatment [4]. Two strategies facilitate early detection: timely detection of symptomatic cancer is crucial in identifying malignancies at their initial stage. Conversely, screening attempts to detect individuals exhibiting particular cancer indications or precancerous conditions without any symptomatic manifestation and promptly refer them for further diagnosis and therapeutic intervention [5]. In addition, Genome‐Wide Association Studies (GWAS) have recognized many loci allied with cancer risk. These loci contain a multitude of Single Nucleotide Polymorphisms (SNPs) that exert regulatory control in gene expression. Consequently, these SNPs can influence an individual's genetic susceptibility to cancer via various mechanisms [6].

In recent decades, GWAS have recognized numerous loci associated with increased risk, encompassing many SNPs [7]. Several SNPs associated with cancer have been identified as having a causal relationship, while in some instances, the functional mechanisms responsible for the association between these SNPs and cancer risk have been elucidated [8, 9]. To date, multiple GWAS have been undertaken over the previous decade to investigate various types of malignancies, including but not restricted to breast, lung, prostate, colorectal, and others [6, 10, 11, 12].

The involvement of inflammation in cancer progression is multifaceted, encompassing various mechanisms such as immune suppression, tissue remodeling, DNA damage, and stimulation of cell proliferation. Chronic inflammation has suppressed the immune response, thereby impeding the identification and subsequent elimination of tumor cells [13]. The inhibitory effect of cytokines secreted by inflammatory cells on the functionality of immune cells facilitates the proliferation and dissemination of cancer cells [14]. IL‐8, also called CXCL8, is a cytokine intricately associated with the inflammatory response. It influences various cellular mechanisms, encompassing the convergence of cancer plasticity, angiogenesis, and immune suppression [15]. Several studies have documented that IL‐8 exhibits increased expression levels in certain tumor cell types, and the upregulation of IL‐8 has been associated with the processes of invasion and metastasis [16]. Cancer susceptibility has been extensively documented in six polymorphisms (−251, −353, +678, +1633, +2767, +781) within the IL‐8 gene.

Despite the existence of multiple meta‐analyses, the current sample size remains insufficient. Therefore, re‐analyzing the association between IL‐8 gene six polymorphisms and the risk of susceptibility for cancer is imperative [17, 18, 19, 20, 21, 22, 23, 24, 25, 26, 27, 28, 29, 30, 31, 32, 33, 34, 35, 36, 37, 38, 39, 40, 41, 42, 43, 44, 45, 46, 47, 48, 49, 50, 51, 52, 53, 54, 55, 56, 57, 58, 59, 60, 61, 62, 63, 64, 65, 66, 67, 68, 69, 70, 71, 72, 73, 74, 75, 76, 77, 78, 79, 80, 81, 82, 83, 84, 85, 86, 87, 88, 89, 90, 91, 92, 93, 94, 95, 96, 97, 98, 99, 100, 101, 102, 103, 104, 105, 106, 107, 108, 109, 110, 111, 112, 113, 114, 115, 116, 117, 118, 119, 120, 121, 122, 123, 124, 125, 126, 127, 128, 129, 130, 131, 132, 133, 134, 135, 136]. Besides, we will evaluate the relationship between IL‐8 expression and gastric cancer based on the TCGA data and our own clinic information.

## 2 Materials and Methods

### 2.1 Bioinformatics Analysis

The differential expression of IL‐8 between various tumor types and adjacent para‐cancerous tissue was examined via data obtained from the Gene Expression Profiling Interactive Analysis (GEPIA) website. The data of overall survival and disease‐free survival concerning the expression of IL‐8 in each tumor were obtained from the above website. The present study investigates the clinical characteristics associated with the expression of IL‐8 and its association with gastric cancer, utilizing data obtained from TCGA database.

### 2.2 Data Eligibility and Credentials of Relevant Studies

Extensive literature was searched in Google Scholar, PubMed, Embase, Web of Science, and Chinese databases. The most recent search was conducted on June 23, 2023. The search strategy used keywords such as “Interleukin‐8,” “IL‐8,” “CXCL8,” “polymorphism,” “variant,” “cancer,” “carcinoma,” and “tumor.” A comprehensive search yielded 973 articles, from which 104 distinct articles met the predefined inclusion criteria. Each type of cancer is diagnosed by clinical pathologists through HE staining or immunohistochemistry. It should be noted that some of these specimens were obtained through puncture, while others were obtained through surgical resection. There are no requirements for the size of tumor tissue or the location of the lesion, as long as which is sufficient for the pathological diagnosis. All cancer patients and their control healthy population were sampled from peripheral blood and tested for SNPs in IL‐8 gene using different detection methods.

### 2.3 Study Criteria

The present analysis incorporated studies that fulfill the following criteria of inclusion: (a) association between cancer susceptibility and just more types of six IL‐8 polymorphisms (−251, −353, +678, +1633, +2767, +781); (b) study design about case–control groups; and (c) adequate availability of each genotype data for both cases and controls or alternatively for certain genetic models; (d) Each type of cancer patients and healthy control population must be informed of the purpose, methods, significance, and risks of the study. Moreover, it is required to fill out a detailed questionnaire, mainly including age, gender, BMI, smoking history, alcohol consumption history, family history of cancer, cancer staging, and so on. The investigation period depends on each type of cancer and should not exceed 1 year at most. Finally, it is necessary to sign the informed consent form for the enrolled population. Also the subsequent exclusion criteria were implemented: First, no control population was included in the analysis, which may have affected the interpretation of the results. Second, the genotype frequency data was unavailable, which could have provided valuable insights into the genetic composition of the study population. Finally, duplicated publications should be identified and deleted.

### 2.4 Data Extraction for Meta‐Analysis

The study encompassed the collection of several key variables, including the name of authors, publication year, country of origin, ethnicity of the participants, specific type of cancer under investigation, the number of cases and controls, source of the control group, assessment of Hardy–Weinberg Equilibrium (HWE) in the control group, and the employed for genotyping.

### 2.5 Data Analysis

The present study measured odds ratios (OR) accompanied by 95% confidence intervals (CI) to evaluate the link between IL‐8 six polymorphisms and cancer risk. This was determined by comparing the genotype frequencies in both groups (cases and controls). The statistical importance of the summary OR was assessed via *Z*‐test [137]. The heterogeneity assumption was determined using a chi‐square‐based *Q*‐test between the studies: a *p* value greater than 0.05 was obtained and the random effects model was employed; however, the fixed effects model was selected [138, 139]. We employed various statistical analyses, including allelic contrast, homozygote comparison, dominant genetic model, heterozygote comparison, and recessive genetic model. The evaluation of HWE was executed in the control group via the Pearson chi‐square test. In order to examine the potential publication bias, Begg's and Egger's tests were conducted [140]. All statistical analyses for this meta‐analysis were conducted via Stata software (Version 11.0; StataCorp LP, College Station, TX). Finally, the quality of studies in meta‐analysis was assessing by Newcastle‐Ottawa Scale method [141].

### 2.6 Information of Participants

In this study, 90 patients were newly diagnosed with gastric cancer from February 2018 to July 2022. These patients were recruited from the Affiliated Hospital of Jiangnan University, and were selected based on clinical signs, tumor location, and tumor grade and stage according to WHO criteria. The histological confirmation of gastric cancer diagnosis was executed by pathologists affiliated with the Department of Pathology at the Affiliated Hospital of Jiangnan University. An age‐matched healthy control group (*n* = 90) was also recruited during the same time period undergoing routine physical examinations in the outpatient. The Each study participant was required to provide a peripheral blood sample of 2 mL. The ethical approval was obtained from the Institutional Review Board (IRB) of the Affiliated Hospital of Jiangnan University. Each participant's written informed consent was also obtained before the sample collection.

### 2.7 Genotyping and Enzyme‐Linked Immunosorbent Assay (ELISA)

For the present study, −251 polymorphism genotypes were assessed with a TaqMan assay using the approach documented by Castro et al. [142]. The levels of IL‐8 in serum were quantified via an ELISA kit (Abcam Co. ltd.). For specific operational procedures and data processing, please refer to previous reference [12].

## 3 Results

### 3.1 Study Selection via Meta‐Analysis

**FIGURE 1:**
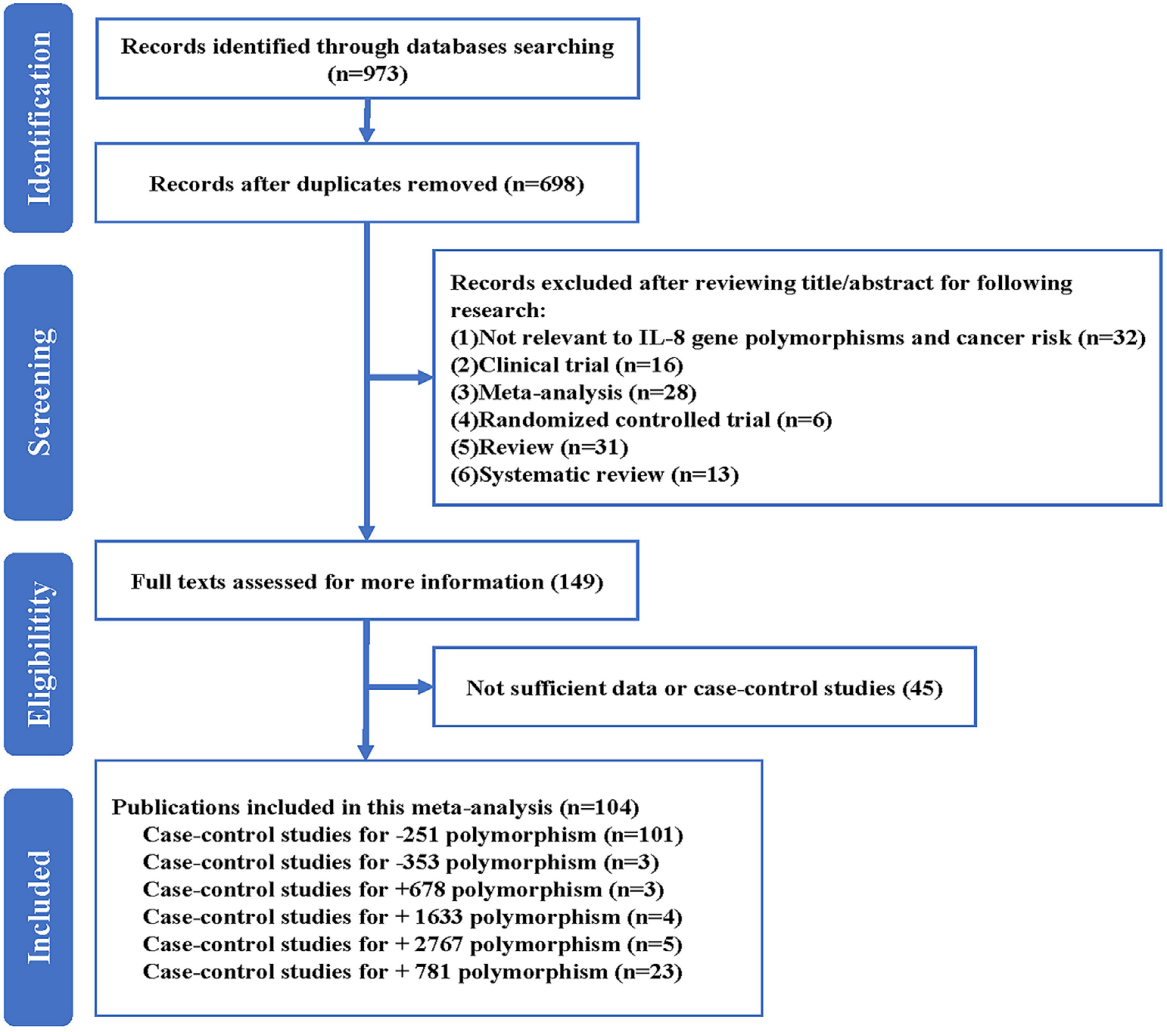
Flowchart depicting the systematic search strategy employed for the identification of studies investigating IL‐8 gene polymorphisms and their potential association with overall cancer risk.

**FIGURE 2:**
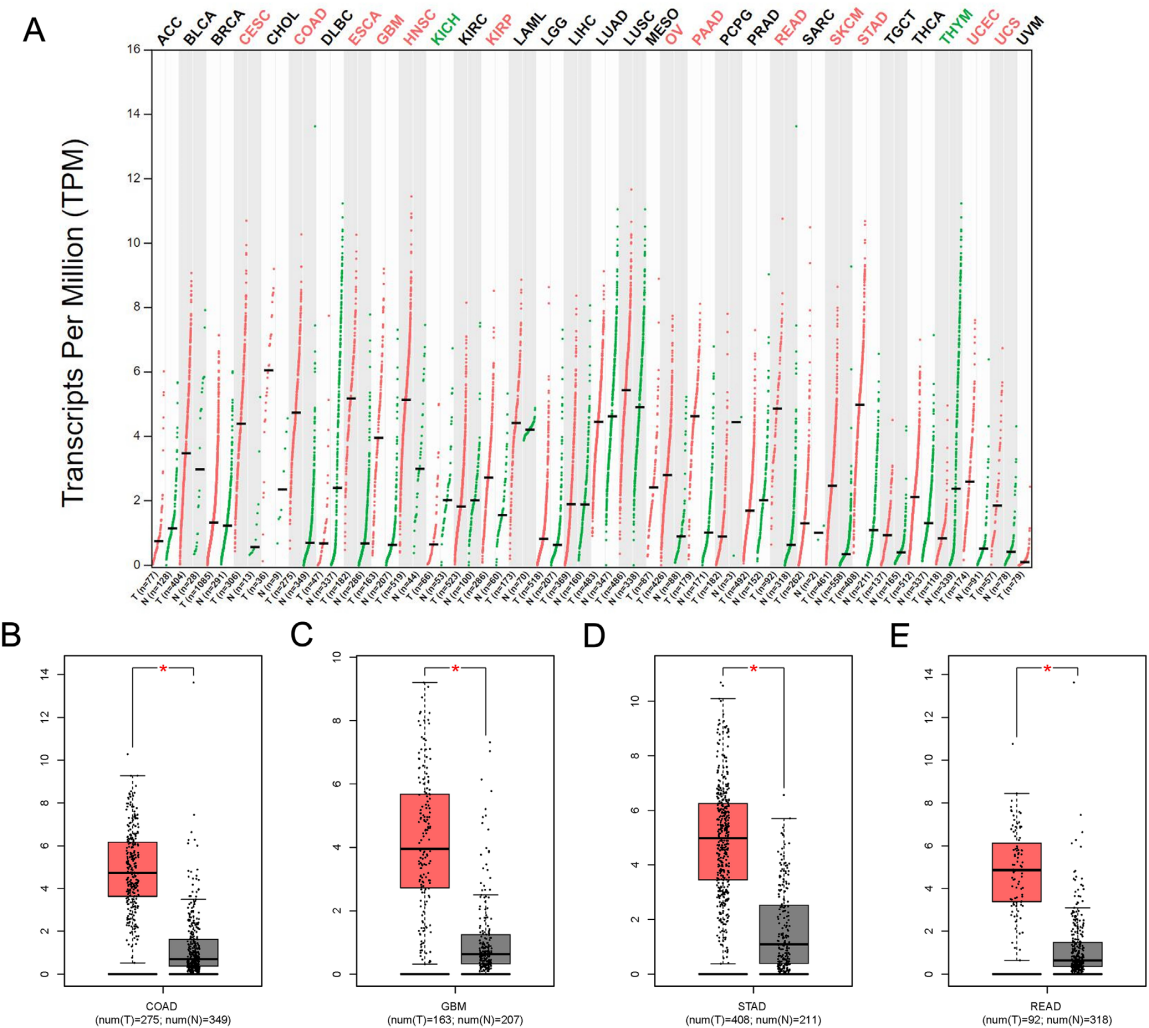
Bioinformatics examinations of IL‐8 gene. (A) The expression profile of IL‐8 gene in all tumor samples and paired normal tissues. (B) IL‐8 gene expression in colon adenocarcinoma. *p < 0.05. (C) IL‐8 gene expression in glioblastoma multiforme. (D) IL‐8 gene expression in stomach adenocarcinoma. (E) IL‐8 gene expression in rectum adenocarcinoma. ACC, adrenocortical carcinoma; BLCA, bladder urothelial carcinoma; BRCA, breast invasive carcinoma; CESC, cervical squamous cell carcinoma and endocervical adenocarcinoma; CHOL, cholangiocarcinoma; COAD, colon adenocarcinoma; DLBC, lymphoid neoplasm diffuse large B‐cell lymphoma; ESCA, esophageal carcinoma; GBM, glioblastoma multiforme; HNSC, head and neck squamous cell carcinoma; KICH, kidney chromophobe; KIRC, kidney renal clear cell carcinoma; KIRP, kidney renal papillary cell carcinoma; LAML, acute myeloid leukemia; LGG, brain lower grade glioma; LIHC, liver hepatocellular carcinoma; LUAD, lung adenocarcinoma; LUSC, lung squamous cell carcinoma; MESO, mesothelioma; OV, ovarian serous cystadenocarcinoma; PAAD, pancreatic adenocarcinoma; PCPG, pheochromocytoma and paraganglioma; PRAD, prostate adenocarcinoma; READ, rectum adenocarcinoma; SARC, sarcoma; SKCM, skin cutaneous melanoma; STAD, stomach adenocarcinoma; TGCT, testicular germ cell tumors; THCA, thyroid carcinoma; THYM, thymoma; UCEC, uterine corpus endometrial carcinoma; UCS, uterine carcinosarcoma; UVM, uveal melanoma.

A comprehensive search of various databases yielded 973 articles. Following a deep evaluation, 104 distinct publications were deemed suitable for inclusion in the present study (Figure 1). The comprehensive details regarding the incorporated studies have been presented in Table 1. The IL‐8 expression was remarkably elevated in tumor tissues in contrast to normal tissues in multiple types of cancer (Figure 2A). This observation is supported by the data presented in Figure 2B–E.

**TABLE 1:** Characteristics of included studies about polymorphisms in IL‐8 gene polymorphisms and cancer risk.

Author	Year	Country	Ethnicity	Cancer type	Case	Control	SOC	HWE	Genotype
−251									
Ahirwar [21]	2010	India	Asian	Bladder cancer	205	270	PB	0.005	AS‐PCR
Smith [100]	2004	UK	Caucasian	Breast cancer	119	235	PB	0.131	ARMS‐PCR
Zhang [127]	2017	China	Asian	Breast cancer	442	447	HB	0.948	PCR‐RFLP
Kamali‐Sarvestani [63]	2007	Iran	Asian	Breast cancer	257	233	HB	0.26	AS‐PCR
Snoussi [102]	2010	Tunisia	African	Breast cancer	409	301	PB	0.173	AS‐PCR
Wang [116]	2022	China	Asian	Breast cancer	1232	1232	HB	0.231	PCR‐RFLP
Wang [119]	2014	China	Asian	Breast cancer	474	501	HB	0.005	PCR‐RFLP
Althubyani [24]	2020	Eygypt	African	Colorectal cancer	70	70	HB	0.932	TaqMan
Burada [33]	2013	Romania	Caucasian	Colorectal cancer	144	233	HB	0.291	TaqMan
Ankathil [25]	2019	Malaysia	Asian	Colorectal cancer	280	280	HB	< 0.001	PCR‐RFLP
Walczak [113]	2012	Poland	Caucasian	Colorectal cancer	191	205	PB	0.001	PCR‐RFLP
Theodoropoulos [107]	2006	Greece	Caucasian	Colorectal cancer	222	196	HB	0.327	PCR‐RFLP
Landi [74]	2003	Spain	Caucasian	Colorectal cancer	352	308	HB	0.047	TaqMan
Basavaraju [27]	2015	USA	Caucasian	Colorectal cancer	388	491	PB	0.711	TaqMan
Küry [73]	2008	France	Caucasian	Colorectal cancer	923	1121	HB	0.033	TaqMan
Tsilidis [108]	2009	USA	Caucasian	Colorectal cancer	205	362	PB	0.058	TaqMan
Mustapha [88]	2012	Malaysia	Asian	Colorectal cancer	255	264	HB	< 0.001	AS‐PCR
Gunter [58]	2006	Italy	Caucasian	Colorectal cancer	205	191	HB	0.84	TaqMan
Wilkening [120]	2008	Sweden	Caucasian	Colorectal cancer	300	580	HB	0.476	TaqMan
Vogel [112]	2007	Denmark	Caucasian	Colorectal cancer	355	753	PB	0.627	PCR‐CE‐SSCP
Malespín‐Bendana [83]	2021	Costa Rica	Mixed	Gastric cancer	46	81	HB	0.907	PCR‐RFLP
Kamali‐Sarvestani [64]	2006	Iran	Asian	Gastric cancer	19	153	HB	0.797	AS‐PCR
Qadri [91]	2014	India	Asian	Gastric cancer	130	200	HB	0.066	PCR‐RFLP
Kamangar [65]	2006	USA	Caucasian	Gastric cancer	112	207	PB	0.055	TaqMan
Felipe [51]	2012	Brasil	Mixed	Gastric cancer	104	196	HB	0.065	PCR‐RFLP
Garza‐Gonzalez [55]	2007	USA	Mixed	Gastric cancer	78	207	HB	0.492	PCR‐RFLP
Ye [124]	2009	Korea	Asian	Gastric cancer	153	206	PB	0.72	PCR‐RFLP
Shirai [133]	2006	Japan	Asian	Gastric cancer	181	468	HB	0.343	PCR‐RFLP
Song [103]	2009	China	Asian	Gastric cancer	125	140	HB	0.72	PCR‐RFLP
Burada [32]	2012	Craiova	Caucasian	Gastric cancer	105	242	HB	0.386	AS‐PCR
Vinagre [111]	2011	Brasil	Mixed	Gastric cancer	102	103	HB	0.15	PCR‐RFLP
Wang [117]	2016	China	Asian	Gastric cancer	132	296	HB	0.946	PCR‐RFLP
Zeng [20]	2005	China	Asian	Gastric cancer	104	94	HB	0.212	PCR‐RDB
Zeng [20]	2005	China	Asian	Gastric cancer	102	102	HB	0.042	PCR‐RDB
Chang [129]	2017	Korea	Asian	Gastric cancer	283	176	HB	0.136	PCR‐RFLP
Chang [129]	2017	Korea	Asian	Gastric cancer	283	284	HB	0.082	PCR‐RFLP
Bo [30]	2010	China	Asian	Gastric cancer	208	190	HB	0.389	PCR‐RFLP
Taguchi [105]	2005	Japan	Asian	Gastric cancer	396	252	HB	0.994	PCR‐RFLP
Oliveira [47]	2015	Brazil	Mixed	Gastric cancer	240	207	HB	0.488	PCR‐RFLP
Kumar [72]	2015	India	Asian	Gastric cancer	200	182	PB	0.801	AS‐PCR
Kang [66]	2009	Korea	Asian	Gastric cancer	334	322	PB	0.226	PCR‐RFLP
Canedo [39]	2008	France	Caucasian	Gastric cancer	333	693	PB	0.459	TaqMan
Lu [82]	2005	China	Asian	Gastric cancer	250	300	PB	0.516	PCR‐DHPLC
Lee [76]	2005	China	Asian	Gastric cancer	461	303	HB	0.184	PCR‐RFLP
Savage [96]	2006	USA	Caucasian	Gastric cancer	287	428	PB	0.391	TaqMan
Zhang [128]	2010	China	Asian	Gastric cancer	519	504	PB	0.754	PCR‐RFLP
Li [79]	2010	China	Asian	Gastric cancer	101	137	HB	0.579	PCR‐DHPLC
Ohyauchi [89]	2005	Japan	Asian	Gastric cancer	212	244	HB	0.847	DS
Ko [69]	2009	Korea	Asian	Gastric cancer	81	589	PB	< 0.001	Snapshot
Crusius [45]	2008	France	Caucasian	Gastric cancer	236	1139	PB	0.705	Real‐Time PCR
Leung [78]	2006	China	Asian	Gastric cancer	83	179	HB	0.638	TaqMan
Szoke [104]	2008	Hungary	Caucasian	Gastric cancer	35	168	HB	0.165	ARMS‐PCR
Ramis [94]	2017	Brazil	Mixed	Gastric cancer	9	38	PB	0.691	PCR‐RFLP
Fu [53]	2016	China	Asian	Glioma	127	284	HB	0.251	PCR‐RFLP
Liu [81]	2015	China	Asian	Glioma	300	300	HB	0.772	PCR‐RFLP
Chien [44]	2011	China	Asian	Hepatocellular carcinoma	131	340	HB	0.445	PCR‐RFLP
Wang [114]	2014	China	Asian	Hepatocellular carcinoma	205	208	HB	0.266	PCR‐RFLP/PCR‐SSP
Liao [17]	2011	China	Asian	Hepatocellular carcinoma	390	150	HB	0.104	PCR‐RFLP
Elsamanoudy [132]	2015	Egypt	African	Hepatocellular carcinoma	112	105	HB	0.551	PCR‐RFLP
Qin [92]	2012	China	Asian	Hepatocellular carcinoma	150	150	HB	0.104	PCR‐RFLP
Lu [19]	2015	China	Asian	Hepatocellular carcinoma	454	446	HB	0.115	PCR‐RFLP
Rafrafi [93]	2013	Tunisia	African	Lung cancer	170	225	PB	0.181	PCR‐RFLP
Yamamoto [122]	2017	Japan	Asian	Lung cancer	462	379	HB	0.939	TaqMan
Campa [38]	2004	Norway	Caucasian	Lung cancer	239	210	PB	0.317	TaqMan
Kaanane [62]	2022	Morocco	African	Lung cancer	150	150	PB	0.169	TaqMan
Bhat [29]	2013	India	Asian	Lung cancer	190	200	HB	< 0.001	PCR‐RFLP
Campa [37]	2005	Germany	Caucasian	Lung cancer	2144	2116	PB	0.203	TaqMan
Vogel [134]	2008	Denmark	Caucasian	Lung cancer	403	744	PB	0.672	PCR‐RFLP
Li [130]	2015	China	Asian	Lung cancer	132	150	HB	0.894	PCR‐HRM
Tai [18]	2007	China	Asian	Nasopharyngeal carcinoma	105	109	HB	0.886	PCR‐RFLP
Huang [61]	2018	China	Asian	Nasopharyngeal carcinoma	176	352	HB	0.109	PCR‐RFLP
Nasr [28]	2007	Tunisia	African	Nasopharyngeal carcinoma	160	169	PB	0.349	PCR‐SSP
Wei [131]	2007	China	Asian	Nasopharyngeal carcinoma	280	290	PB	0.164	PCR‐RFLP
Matos [46]	2019	Brazil	Mixed	Oral cancer	66	130	HB	0.493	PCR
Vairaktaris [110]	2007	Germany	Caucasian	Oral cancer	158	156	HB	< 0.001	PCR‐RFLP
Qin [92]	2012	China	Asian	Oral cancer	150	150	HB	0.104	PCR‐RFLP
Liu [82]	2012	China	Asian	Oral cancer	270	350	HB	0.454	PCR‐RFLP
Singh [99]	2016	India	Asian	Oral cancer	300	300	HB	< 0.001	PCR‐RFLP
Campa [36]	2017	Germany	Caucasian	Oral cancer	153	725	HB	0.524	TaqMan
Shimizu [98]	2008	Japan	Asian	Oral cancer	69	91	HB	0.296	PCR‐FLP
Kietthubthew [67]	2010	Thailand	Asian	Oral cancer	63	99	PB	0.813	TaqMan
Moreno‐Guerrero [87]	2021	Mexico	Mixed	Neuroblastoma	27	38	HB	0.152	PCR‐RFLP
Kilic [68]	2016	Turkey	Caucasian	Thyroid cancer	101	109	HB	0.586	PCR
Wu [121]	2013	China	Asian	Urothelial carcinoma	300	594	HB	0.075	PCR‐RFLP
Kuyl [135]	2004	Netherlands	Caucasian	Kaposi's sarcoma	84	153	HB	0.382	PCR‐RFLP
Chen [43]	2016	China	Asian	Osteosarcoma	190	190	HB	< 0.001	PCR‐RFLP
Howell [59]	2003	UK	Caucasian	Melanoma	142	233	HB	0.16	ARMS–PCR
Cacev [35]	2008	Croatia	Caucasian	Colon cancer	160	160	PB	0.346	PCR‐RFLP
Koensgen [70]	2014	Germany	Caucasian	Ovarian cancer	267	426	HB	0.026	PCR‐RFLP
Franz [52]	2017	Brazil	Mixed	Prostate cancer	175	185	HB	0.127	PCR‐SSP
Chen [42]	2016	China	Asian	Prostate cancer	439	524	HB	0.129	PCR‐RFLP
Taheri [106]	2019	Iran	Asian	Prostate cancer	355	200	HB	0.689	ARMS‐PCR
Yang [123]	2006	USA	Caucasian	Prostate cancer	520	418	PB	0.168	ht‐SNP
McCarron [136]	2002	UK	Caucasian	Prostate cancer	238	235	HB	0.131	PCR
Michaud [85]	2006	USA	Caucasian	Prostate cancer	484	613	PB	0.777	PCR
Leibovici [77]	2005	USA	Caucasian	Bladder cancer	463	440	HB	NA	TaqMan
Zamora‐Ros [125]	2015	Spain	Caucasian	Colorectal cancer	344	303	HB	NA	TaqMan
Oliveira [48]	2013	Brazil	Mixed	Gastric cancer	200	240	HB	NA	PCR‐RFLP
Pan [90]	2014	China	Asian	Gastric cancer	308	308	PB	NA	MALDI‐TOF MS
Boonyanugomol [31]	2019	Korea	Asian	Gastric cancer	10	72	HB	NA	PCR‐RFLP
Zhang [126]	2010	USA	Caucasian	Prostate cancer	162	173	PB	NA	MOLD‐TOF‐MS
−353									
Wei [131]	2007	China	Asian	Nasopharyngeal carcinoma	280	290	PB	0.406	PCR‐RFLP
Wang [114]	2014	China	Asian	Hepatocellular carcinoma	205	208	HB	0.474	PCR‐RFLP/PCR‐SSP
Zhang [127]	2017	China	Asian	Breast cancer	442	447	HB	< 0.001	PCR‐RFLP
+678									
Wei [131]	2007	China	Asian	Nasopharyngeal carcinoma	280	290	PB	0.064	PCR‐RFLP
Ahirwar [21]	2010	India	Asian	Bladder cancer	205	270	PB	< 0.001	AS‐PCR
Wang [114]	2014	China	Asian	Hepatocellular carcinoma	205	208	HB	0.161	AS‐PCR
+1633									
Chien [44]	2011	China	Asian	Hepatocellular carcinoma	131	340	HB	0.562	PCR‐RFLP
Liu [82]	2012	China	Asian	Oral cancer	270	350	HB	0.569	PCR‐RFLP
Koensgen [70]	2014	Germany	Caucasian	Ovarian cancer	246	62	HB	0.865	PCR‐RFLP
Huang [61]	2018	China	Asian	Nasopharyngeal carcinoma	176	352	HB	0.109	PCR‐RFLP
+2767									
Chien [44]	2011	China	Asian	Hepatocellular carcinoma	131	340	HB	0.392	PCR‐RFLP
Liu [82]	2012	China	Asian	Oral cancer	270	350	HB	0.029	PCR‐RFLP
Koensgen [70]	2014	Germany	Caucasian	Ovarian cancer	268	426	HB	0.029	PCR‐RFLP
Hsieh [60]	2007	China	Asian	Leiomyoma	162	156	HB	0.078	PCR‐RFLP
Huang [61]	2018	China	Asian	Nasopharyngeal carcinoma	176	352	HB	0.012	PCR‐RFLP
−781									
Liu [81]	2015	China	Asian	Glioma	300	300	HB	0.049	PCR‐RFLP
Kamangar [65]	2006	USA	Caucasian	Gastric cancer	111	208	PB	0.158	TaqMan
Bo [30]	2010	China	Asian	Gastric cancer	208	190	HB	0.225	PCR‐RFLP
Chien [44]	2011	China	Asian	Hepatocellular carcinoma	131	340	HB	0.776	PCR‐RFLP
Liu [82]	2012	China	Asian	Oral cancer	270	350	HB	0.781	PCR‐RFLP
Qin [92]	2012	China	Asian	Oral cancer	150	150	HB	0.041	PCR‐RFLP
Rafrafi [93]	2013	Tunisia	African	Lung cancer	170	225	PB	0.329	PCR‐RFLP
Wang [114]	2014	China	Asian	Hepatocellular carcinoma	205	208	HB	0.549	PCR‐RFLP/PCR‐SSP
Koensgen [70]	2014	Germany	Caucasian	Ovarian cancer	267	426	HB	0.1	PCR‐RFLP
Chen [43]	2016	China	Asian	Osteosarcoma	190	190	HB	0.116	PCR‐RFLP
Taheri [106]	2019	Iran	Asian	Prostate cancer	355	200	HB	0.639	ARMS‐PCR
Kaanane [62]	2022	Morocco	African	Lung cancer	150	150	PB	0.307	TaqMan
Alkanli [23]	2023	Turkey	Caucasian	Bladder cancer	88	89	HB	0.608	PCR‐RFLP
Moreno‐Guerrero [87]	2021	Mexico	Mixed	Neuroblastoma	27	38	HB	0.313	PCR‐RFLP
Song [103]	2009	China	Asian	Gastric cancer	125	140	HB	0.48	PCR‐RFLP
Fu [53]	2016	China	Asian	Glioma	127	284	HB	0.788	PCR‐RFLP
Zhang [127]	2017	China	Asian	Breast cancer	442	447	HB	0.327	PCR‐RFLP
Huang [61]	2018	China	Asian	Nasopharyngeal carcinoma	176	352	HB	0.671	PCR‐RFLP
Ghazy [56]	2021	Saudi Arabia	Asian	Prostate cancer	40	40	HB	0.673	real‐time PCR
Liao [17]	2011	China	Asian	Hepatocellular carcinoma	150	150	HB	0.041	PCR‐RFLP
Elsamanoudy [132]	2015	Egypt	African	Hepatocellular carcinoma	112	105	HB	0.178	PCR‐RFLP
Qin [92]	2012	China	Asian	Hepatocellular carcinoma	150	150	HB	0.041	PCR‐RFLP
Lu [19]	2015	China	Asian	Hepatocellular carcinoma	454	446	HB	0.062	PCR‐RFLP

Abbreviations: ARMS: amplification refractory mutation system; AS: allele specific primer; CE‐SSCP: capillary electrophoresis‐single strand conformation polymorphism; DHPLC: denaturing high performance liquid chromatography; HB: hospital‐based; HRM: high resolution melt; HWE: Hardy–Weinberg equilibrium of control group; MALDI‐TOF MS: matrix‐assisted laser desorption/ionization time of flight mass spectrometry; PB: population‐based; PCR‐RFLP: polymerase chain reaction followed by restriction fragment length polymorphism; SOC; source of control; SSP: sequence specific primer.

### 3.2 Relationship Between the Expression of IL‐8 and Gastric Cancer From TCGA Data

**FIGURE 3:**
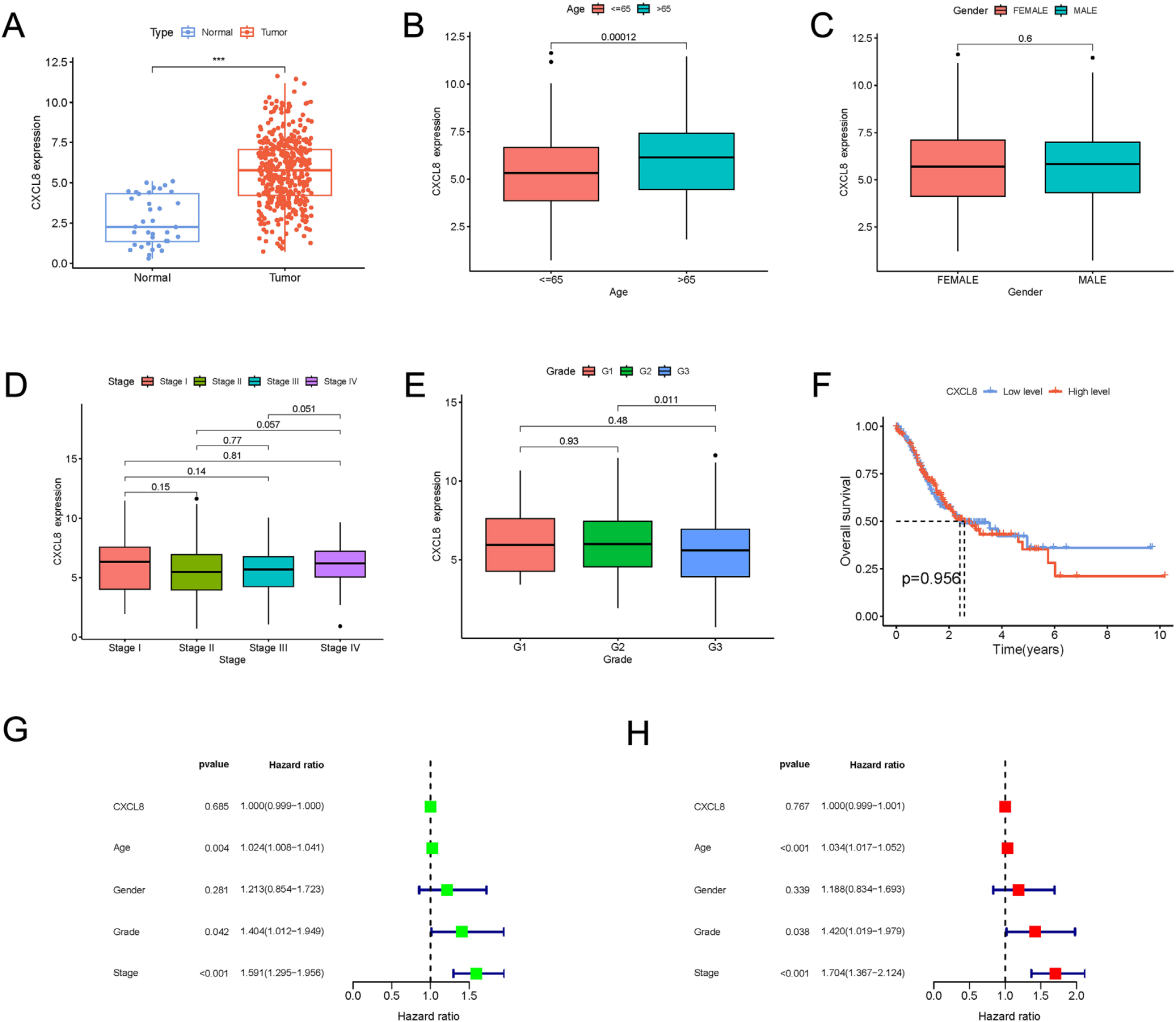
TCGA database information shows IL‐8 expression in gastric cancer. (A) The expression of the IL‐8 gene in samples of gastric carcinoma and paired normal tissues. (B) The expression of the IL‐8 gene in gastric cancer samples from individuals less than 65 years and older than 65 years. (C) The IL‐8 gene expression difference between female and male gastric cancer samples. (D) The expression of IL‐8 at various gastric cancer stages. (E) The expression of IL‐8 among different grades of gastric cancer. (F) The overall survival between the level of IL‐8 gene expression. Analyses of prognostic factors for progression‐free survival in univariate (G) and multivariate (H).

First, the expression of IL‐8 was remarkably elevated in tumor tissue compared to normal tissue (*p* < 0.001) (Figure 3A). Second, clinicopathological factors of gastric cancer were analyzed, age more than 65 had higher expression of IL‐8 (*p* < 0.05) (Figure 3B), however, no positive result was observed in subgroup including gender, grade and TNM stage (Table 2) (Figure 3C–F). Furthermore, prognostic factors for recurrence‐free survival were calculated using univariate and multivariate analyses. Age, grade and stage were three significant prognostic factors (*p* < 0.05) (Table 3) (Figure 3G,H).

**TABLE 2:** Correlation between IL‐8 expression and clinicopathological factors in gastric cancer from TCGA database.

Covariates	Group	Total	IL‐8 expression		chi	*p*
			Low	High		
Age	≤ 65	184 (45.21%)	110 (53.66%)	74 (36.63%)	11.228	8.00E‐04
	> 65	223 (54.79%)	95 (46.34%)	128 (63.37%)		
Gender	Female	145 (35.19%)	74 (35.92%)	71 (34.47%)	0.0426	0.8365
	Male	267 (64.81%)	132 (64.08%)	135 (65.53%)		
	G1	12 (2.98%)	6 (2.99%)	6 (2.97%)	2.0173	0.3647
	G2	148 (36.72%)	67 (33.33%)	81 (40.1%)		
	G3	243 (60.3%)	128 (63.68%)	115 (56.93%)		
Grade	I	58 (14.95%)	25 (12.76%)	33 (17.19%)	5.0362	0.1692
	II	122 (31.44%)	68 (34.69%)	54 (28.12%)		
	III	169 (43.56%)	88 (44.9%)	81 (42.19%)		
	IV	39 (10.05%)	15 (7.65%)	24 (12.5%)		
T	T1	22 (5.45%)	11 (5.37%)	11 (5.53%)	4.0643	0.2546
	T2	88 (21.78%)	38 (18.54%)	50 (25.13%)		
	T3	181 (44.8%)	101 (49.27%)	80 (40.2%)		
	T4	113 (27.97%)	55 (26.83%)	58 (29.15%)		
M	M0	365 (93.35%)	186 (94.42%)	179 (92.27%)	0.4218	0.516
	M1	26 (6.65%)	11 (5.58%)	15 (7.73%)		
N	N0	124 (31.55%)	62 (31.31%)	62 (31.79%)	1.0709	0.7841
	N1	109 (27.74%)	51 (25.76%)	58 (29.74%)		
	N2	78 (19.85%)	41 (20.71%)	37 (18.97%)		
	N3	82 (20.87%)	44 (22.22%)	38 (19.49%)		

**TABLE 3:** Prognostic factors for recurrence‐free survival in univariate and multivariate analyses.

Covariates	Univariate analysis	*p*	Multivariate	*p*
	HR (95%CI)		HR (95%CI)	
CXCL8	0.999 (0.999–1)	0.684	0.999 (0.999–1)	0.766
Age	1.024 (1.007–1.041)	0.003	1.034 (1.016–1.052)	0
Gender	1.212 (0.854–1.722)	0.28	1.188 (0.834–1.692)	0.339
Grade	1.404 (1.011–1.949)	0.0423	1.419 (1.018–1.978)	0.038
Stage	1.591 (1.294–1.956)	1.03E‐05	1.703 (1.366–2.123)	2.16E‐06

### 3.3 Meta‐Analysis

**FIGURE 4:**
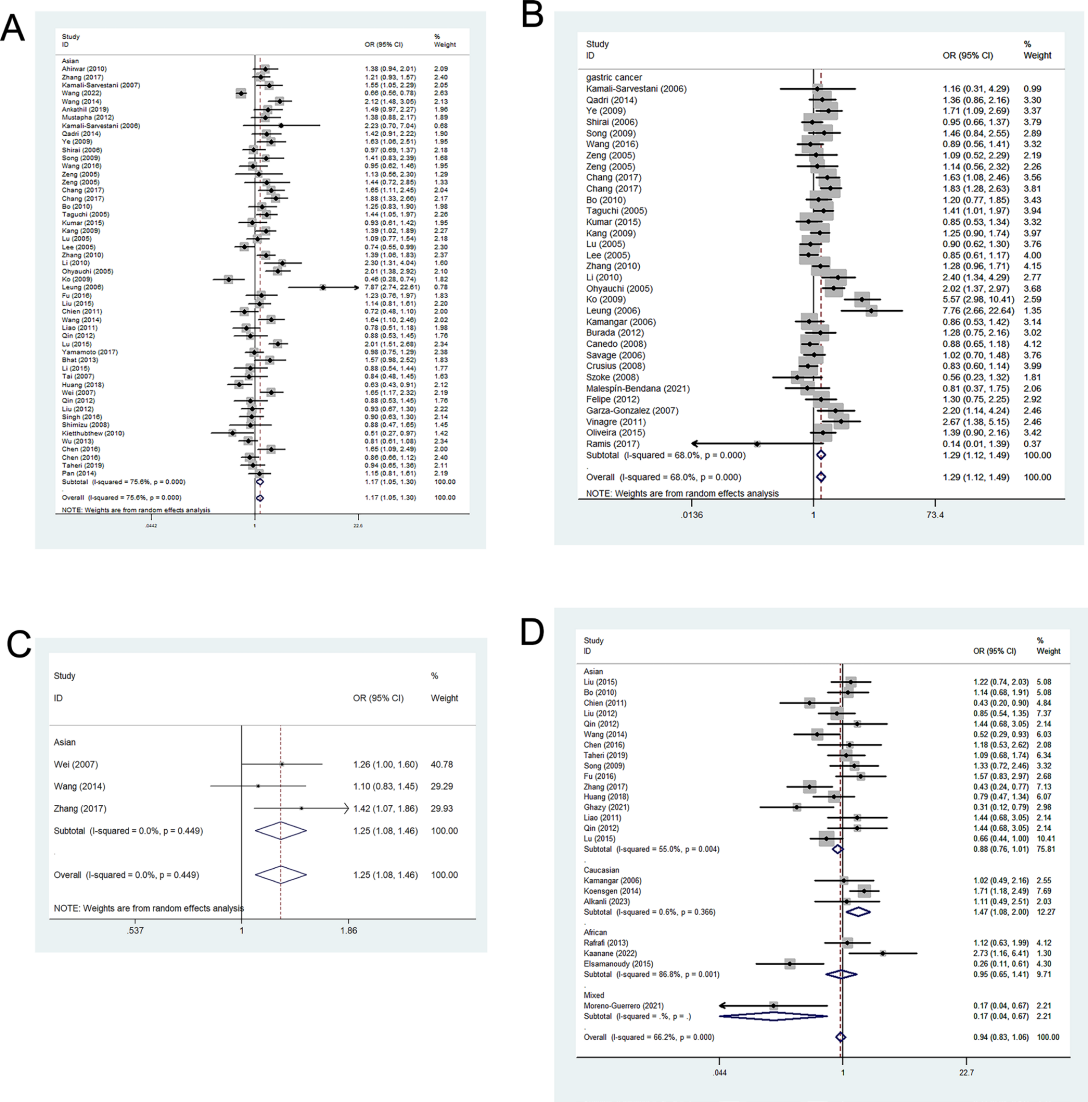
Forest plots corresponding to cancer‐related risk between the IL‐8 polymorphisms. The squares and horizontal lines respectively correspond to the study‐specific ORs and 95% CIs, with square area being indicative of weight (the inverse of the variance). Diamonds additionally reflect the summary OR and 95% CI. (A) Relationship between −251 polymorphism and cancer risk in Asians based on the dominant genetic model. (B) Relationship between −251 polymorphism and gastric cancer based on heterozygote comparison. (C) Relationship between −353 polymorphism and cancer risk based on allelic contrast. (D) Relationship between +781 polymorphism and cancer risk in Caucasians based on the recessive genetic model.

The analysis revealed a significant increase in the correlation among the −251 polymorphism and cancer risk (such as: A‐allele vs. T‐allele, OR = 1.078, 95%CI = 1.020–1.140, *p* = 0.008, Table 4). Furthermore, a higher prevalence of associations was observed among Asian in relation to the −251 polymorphism (such as: AA+AT vs. TT: OR = 1.168, 95%CI = 1.047–1.303, *p* < 0.005, Figure 4A, Table 4). Substantial relationships in four distinct types of cancer were observed (such as gastric cancer: such as AT vs. TT, OR = 1.292, 95%CI = 1.117–1.494, *p* = 0.001, Figure 4B, Table 4). There was an elevated link between −353 polymorphism and cancer risk, such as A‐allele versus T‐allele, OR = 1.255, 95%CI = 1.079–1.459, *p* = 0.003 (Figure 4C, Table 4). For −781 polymorphism, a single potential link was noted in the ethnicity subgroup: Caucasian, TT versus TC + CC, OR = 1.472, 95%CI = 1.078–2.009, *p* = 0.015 (Figure 4D, Table 4).

**TABLE 4:** Stratified analyses of *IL‐8* genes common polymorphisms on cancer risk.

Variables	No	Case/Controls	M‐allele versus W‐allele OR (95%CI) *P*_h_ *P*	MM versus WW OR (95%CI) *P*_h_ *P*	MW versus WW OR (95%CI) *P*_h_ *P*	MM + MW versus WW OR (95%CI) *P*_h_ *P*	MM versus MW + WW OR (95%CI) *P*_h_ *P*
IL‐8 −251							
Total	101	25 750/31995	1.078 (1.020–1.140)0.000 0.008	1.171 (1.052–1.303)0.000 0.004	1.119 (1.033–1.211)0.000 0.006	1.111 (1.032–1.196)0.000 0.005	1.100 (1.004–1.206)0.000 0.041
Ethnicity							
Asian	52	13 058/14784	1.118 (1.022–1.222)0.000 0.014	1.295 (1.088–1.541)0.000 0.004	1.167 (1.037–1.314)0.000 0.010	1.168 (1.047–1.303)0.000 0.005	1.211 (1.031–1.423)0.000 0.020
Caucasian	33	10 574/14766	1.030 (0.969–1.095)0.000 0.342	1.043 (0.930–1.171)0.004 0.469	1.016 (0.907–1.139)0.000 0.780	1.023 (0.923–1.134)0.000 0.664	1.026 (0.948–1.111)0.125 0.524
African	6	1071/1020	0.979 (0.660–1.453)0.000 0.917	0.947 (0.458–1.960)0.000 0.884	1.013 (0.657–1.563)0.004 0.952	0.981 (0.578–1.663)0.000 0.942	0.962 (0.577–1.606)0.000 0.883
Mixed	10	1047/1425	1.076 (0.876–1.321)0.023 0.483	1.093 (0.737–1.619)0.047 0.659	1.402 (1.041–1.889)0.086 0.026	1.205 (0.907–1.602)0.019 0.198	0.874 (0.646–1.183)0.122 0.384
Cancer type							
Gastric cancer	36	6562/9650	1.115 (0.988–1.257)0.000 0.077	1.248 (1.004–1.553)0.000 0.046	1.292 (1.117–1.494)0.000 0.001	1.200 (1.054–1.368)0.000 0.006	1.135 (0.921–1.398)0.000 0.234
Hepatocellular carcinoma	6	1442/1399	1.023 (0.844–1.241)0.014 0.814	0.963 (0.690–1.344)0.096 0.825	1.209 (0.793–1.842)0.000 0.377	1.149 (0.779–1.693)0.000 0.484	0.828 (0.647–1.060)0.255 0.134
Prostate cancer	7	2373/2348	0.958 (0.862–1.064)0.200 0.422	0.936 (0.753–1.163)0.168 0.549	0.929 (0.770–1.119)0.121 0.436	0.927 (0.782–1.099)0.143 0.383	0.936 (0.783–1.120)0.151 0.471
Oral cancer	8	1229/2001	1.075 (0.882–1.311)0.003 0.473	1.206 (0.743–1.960)0.001 0.448	0.863 (0.674–1.104)0.055 0.240	0.965 (0.803–1.160)0.265 0.703	1.369 (0.777–2.414)0.000 0.277
Lung cancer	8	3890/4174	0.888 (0.757–1.042)0.000 0.147	0.830 (0.617–1.115)0.001 0.215	0.926 (0.794–1.080)0.174 0.326	0.889 (0.728–1.086)0.010 0.251	0.861 (0.685–1.083)0.003 0.201
Glioma	2	427/584	1.278 (1.067–1.532)0.406 0.008	1.660 (1.162–2.371)0.315 0.005	0.982 (0.729–1.322)0.386 0.904	1.171 (0.889–1.544)0.817 0.262	1.581 (0.929–2.691)0.085 0.092
Bladder cancer	2	668/710	1.590 (1.227–2.061)0.000 0.000	2.196 (1.377–3.504)0.000 0.001	0.977 (0.635–1.503)0.000 0.917	1.264 (0.989–1.616)0.563 0.061	2.221 (1.463–3.372)0.000 0.000
Breast cancer	6	2933/2949	1.158 (0.885–1.514)0.000 0.285	1.406 (0.829–2.386)0.000 0.206	1.233 (0.831–1.829)0.000 0.299	1.283 (0.845–1.948)0.000 0.242	1.199 (0.868–1.655)0.000 0.271
Colorectal cancer	14	4234/5357	1.121 (0.999–1.257)0.000 0.053	1.320 (1.016–1.715)0.000 0.038	1.101 (0.876–1.385)0.000 0.409	1.107 (0.898–1.364)0.000 0.342	1.230 (1.018–1.486)0.001 0.032
Nasopharyngeal carcinoma	4	721/920	1.079 (0.717–1.623)0.000 0.715	1.131 (0.535–2.392)0.000 0.746	1.116 (0.703–1.772)0.005 0.640	1.124 (0.658–1.920)0.000 0.670	1.075 (0.646–1.789)0.018 0.780
Other cancer	8	1271/1903	1.089 (0.905–1.310)0.011 0.367	1.130 (0.816–1.565)0.087 0.461	1.096 (0.875–1.372)0.102 0.424	1.122 (0.879–1.433)0.026 0.356	1.066 (0.830–1.370)0.225 0.617
Source of control							
HB	70	16 121/19095	1.116 (1.048–1.188)0.000 0.971	1.244 (1.090–1.420)0.000 0.001	1.149 (1.040–1.269)0.000 0.006	1.157 (1.054–1.269)0.000 0.002	1.154 (1.032–1.291)0.000 0.012
PB	31	9629/12900	0.998 (0.894–1.114)0.000 0.001	1.039 (0.863–1.251)0.000 0.685	1.059 (0.925–1.212)0.000 0.405	1.024 (0.907–1.157)0.000 0.698	1.002 (0.851–1.180)0.000 0.983
IL‐8 −353	3	927/945	1.255 (1.079–1.459)0.449 0.003	1.463 (1.068–2.004)0.653 0.018	1.269 (0.980–1.643)0.732 0.070	1.339 (1.052–1.705)0.524 0.018	1.297 (1.031–1.632)0.784 0.026
IL‐8 + 678	3	690/768	1.020 (0.866–1.201)0.970 0.816	1.166 (0.837–1.623)0.814 0.364	0.881 (0.696–1.115)0.390 0.291	0.952 (0.770–1.178)0.785 0.652	1.203 (0.875–1.655)0.702 0.256
IL‐8 + 1633	4	823/1104	0.968 (0.804–1.166)0.166 0.733	0.937 (0.651–1.349)0.197 0.727	0.978 (0.789–1.211)0.569 0.837	0.963 (0.769–1.206)0.304 0.740	0.935 (0.719–1.215)0.372 0.614
IL‐8 + 2767	5	1007/1624	0.930 (0.799–1.082)0.149 0.347	0.875 (0.626–1.224)0.094 0.435	0.924 (0.774–1.102)0.577 0.380	0.913 (0.774–1.078)0.532 0.283	0.905 (0.638–1.283)0.034 0.575
IL‐8 + 781							
Total	23	4398/5178	0.942 (0.836–1.062)0.000 0.329	0.904 (0.694–1.178)0.000 0.454	0.966 (0.846–1.104)0.003 0.617	0.953 (0.824–1.102)0.000 0.518	0.917 (0.733–1.146)0.000 0.446
Ethnicity							
Asian	16	3473/3937	0.948 (0.854–1.054)0.005 0.323	0.884 (0.692–1.131)0.003 0.327	0.969 (0.841–1.117)0.021 0.667	0.956 (0.833–1.098)0.013 0.523	0.893 (0.715–1.116)0.004 0.321
Caucasian	3	466/723	1.189 (0.856–1.653)0.043 0.302	1.528 (0.773–3.020)0.063 0.223	1.225 (0.761–1.973)0.067 0.404	1.276 (0.746–2.181)0.024 0.374	1.472 (1.078–2.009)0.366 0.015
African	3	432/480	0.826 (0.406–1.681)0.000 0.598	0.809 (0.199–3.297)0.000 0.767	0.746 (0.461–1.205)0.070 0.231	0.750 (0.370–1.521)0.001 0.426	0.932 (0.282–3.077)0.001 0.907
Mixed	1	27/38	0.424 (0.208–0.866)0.000 0.018	0.164 (0.033–0.810)0.000 0.027	0.933 (0.271–3.209)0.000 0.913	0.536 (0.167–1.718)0.000 0.294	0.172 (0.044–0.671)0.000 0.011
Cancer type							
Gastric cancer	3	444/538	1.119 (0.931–1.346)0.456 0.232	1.267 (0.864–1.859)0.675 0.226	1.118 (0.845–1.479)0.411 0.434	1.148 (0.883–1.494)0.368 0.303	1.171 (0.826–1.659)0.859 0.375
Hepatocellular carcinoma	6	1202/1399	0.778 (0.595–1.019)0.000 0.068	0.602 (0.357–1.017)0.004 0.058	0.826 (0.575–1.187)0.001 0.301	0.763 (0.529–1.100)0.000 0.147	0.659 (0.415–1.045)0.011 0.076
Prostate cancer	2	395/240	0.613 (0.183–2.056)0.001 0.428	0.352 (0.026–4.717)0.003 0.431	0.535 (0.089–3.207)0.031 0.494	0.429 (0.048–3.804)0.006 0.447	0.622 (0.183–2.114)0.018 0.447
Oral cancer	2	420/500	0.877 (0.724–1.063)0.470 0.181	0.866 (0.548–1.368)0.289 0.536	0.765 (0.579–1.012)0.956 0.061	0.783 (0.600–1.021)0.792 0.070	1.014 (0.627–1.639)0.247 0.955
Lung cancer	2	320/375	1.181 (0.819–1.704)0.127 0.373	1.617 (0.650–4.022)0.083 0.302	0.939 (0.674–1.306)0.791 0.707	1.065 (0.785–1.443)0.363 0.687	1.650 (0.694–3.924)0.089 0.257
Glioma	2	427/584	1.117 (0.883–1.414)0.235 0.355	1.343 (0.888–2.031)0.366 0.163	0.990 (0.728–1.346)0.270 0.949	1.069 (0.780–1.463)0.228 0.680	1.346 (0.906–2.002)0.543 0.142
Other cancer	6	1190/1542	0.976 (0.747–1.274)0.000 0.856	0.858 (0.426–1.727)0.000 0.667	0.980 (0.868–1.107)0.096 0.358	1.080 (0.790–1.477)0.009 0.629	0.801 (0.451–1.422)0.000 0.448
Source of control							
HB	20	3967/4595	0.920 (0.806–1.050)0.000 0.215	0.849 (0.635–1.135)0.000 0.268	0.971 (0.834–1.130)0.001 0.701	0.942 (0.798–1.110)0.000 0.474	0.863 (0.678–1.099)0.000 0.232
PB	3	431/583	1.087 (0.846–1.397)0.191 0.514	1.336 (0.735–2.428)0.147 0.343	0.911 (0.693–1.198)0.919 0.505	1.002 (0.776–1.294)0.512 0.987	1.383 (0.799–2.393)0.170 0.247

Abbreviations: HB: hospital‐based; *P*: *Z*‐test for the statistical significance of the OR; PB: population‐based; *P*_h_: value of *Q*‐test for heterogeneity test; SOC; source of control.

### 3.4 IL‐8 Expression in the Serum of Gastric Cancer Patients

**FIGURE 5:**
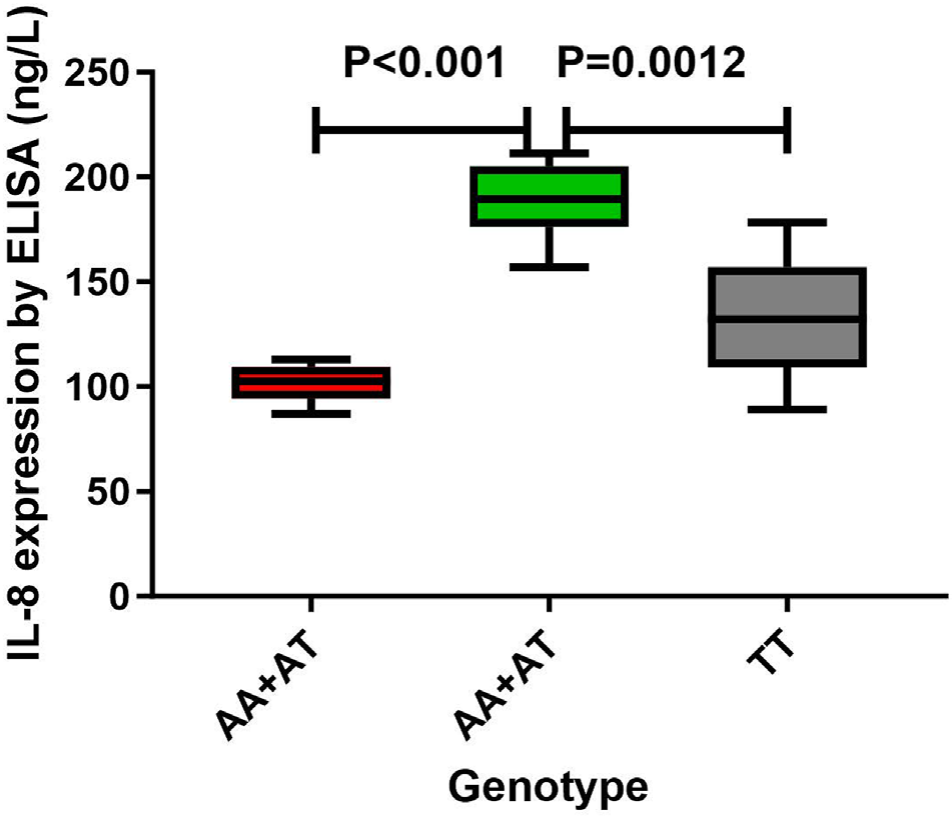
Serum analysis of IL‐8 levels in −251 genotype of gastric cancer using mean values (horizontal lines, mean values). Serum IL‐8 levels in gastric cancer patients carrying AA/TT genotypes were remarkably higher than that carrying TT genotypes (*p* < 0.01). Serum IL‐8 concentrations were also significantly higher in gastric cancer patients with the AA/TT genotypes as compared to healthy controls with the same genotypes (*p* < 0.01).

In this study, 180 serum samples (90 patients were newly diagnosed with gastric cancer and 90 individuals were from age‐matched healthy control group) were collected. These samples were specifically selected to represent various genotypes of the IL‐8 −251 variant via ELISA. Specifically, this study exhibited that the serum IL‐8 levels in gastric cancer patients with AA/TT genotypes were significantly higher than those with TT genotypes and also higher than those with same AA/TT genotypes from healthy controls (*p* < 0.01, as illustrated in Figure 5).

## 4 Discussion

Globally, cancer remains a predominant cause of both mortality and morbidity, resulting in approximately 9 million deaths annually [143]. IL‐8 is a prominent pro‐inflammatory mediator that has been extensively studied as a potential risk factor in the pathogenesis and progression of cancer. In addition, a number of SNPs within the IL‐8 gene, situated in its promoter region, have been implicated in the modulation of IL‐8 expression levels. For example, the A allele of the −251 SNP has been found to be associated with increased protein expression compared to the T allele. Therefore, it is hypothesized that the presence of SNPs in the IL‐8 gene may indirectly influence the expression of IL‐8, potentially influencing the development and progression of tumors [144].

In previous studies, a number of meta‐analyses have been conducted this association, however, the conclusion remains not clear and definite. For instance, Farbod et al. discovered that the IL‐8 −251 T/A polymorphism exhibited a significant association with susceptibility to breast cancer [145]. Additional, Chen et al. proposed that −251 polymorphism of the IL‐8 exhibited a significant association with susceptibility to prostate cancer. Moreover, Wang et al. suggested that this specific polymorphism could potentially act as a genetic biomarker for the identification of gastric cancer in Asian individuals [146]. On the other hand, Rezaei, Antikchi, and Gao et al. reported negative results regarding several types of cancer [147, 148, 149]. Therefore, it is necessary to make an up‐dated analysis. In our current investigation, a comprehensive meta‐analysis was executed to elucidate the potential correlation between six IL‐8 polymorphisms and the susceptibility to various types of cancer, which had two advantages: on one hand, current study included the most largest samples than previous meta‐analysis, on the other hand, serum IL‐8 expression was added, and was analyzed the relationship between different genotypes and IL‐8 expression, which was the novel exploration. Finally, 104 case–control studies were incorporated into the analysis. The findings exhibited a statistically substantial correlation between the IL‐8 −251 polymorphism and susceptibility to various types of cancer. A stratified analysis examined the connection between the −251 polymorphism and various cancer types. The findings revealed that the −251 polymorphism was identified as a risk factor for gastric, glioma, bladder, and colorectal cancer. Specifically, individuals carrying the A‐allele were more susceptible to developing these cancer types. However, no substantial correlation was detected between the −251 polymorphism and the incidence of hepatocellular carcinoma, prostate cancer, or oral cancer. The differential impact of a shared gene polymorphism across various cancer types can be attributed to several factors. First, the etiology of various cancer exhibits considerable heterogeneity. Second, it has been observed that the identical polymorphism of a specific gene exhibits distinct functions in the initiation and progression of diverse tumor types. Third, it is noteworthy that the target sites of gene polymorphism exhibit variations across different tumor types. Fourth, the multifaceted functionality of the same gene polymorphism site acted throughout distinct stages of disease progression. The subgroup analysis based on ethnicity indicates a significant association between the −251 polymorphism and an elevated risk of cancer, specifically in Asians. However, this association was not observed in Caucasians, Africans, or Mixed populations. Furthermore, variant genotypes at −353 were significantly correlated with an elevated susceptibility to cancer. Finally, individuals carrying the +781 A allele may exhibit an increased susceptibility to cancer risk within the Caucasian population.

The incidence of gene polymorphisms exhibits significant variation across diverse ethnic populations, thereby signifying a crucial property of these genetic variations due to following two primary factors: genetic disparities and environmental variations. Ethnic groups inherently possess distinct genetic and environmental backgrounds contributing to the observed differences. Additionally, diverse populations often exhibit dissimilar patterns of linkage disequilibrium, further contributing to the observed variations. Polymorphism has the potential to exhibit close linkage with distinct nearby causal variants across diverse populations.

In this study, a significant discovery was indicated that gastric cancer patients with AA/AT genotypes exhibited elevating levels of IL‐8 expression than healthy controls, suggesting this may offer a valuable biomarker for the early detection for gastric cancer. These results imply that such individuals may be more susceptible to gastric cancer development. Consequently, it is crucial to closely monitor these individuals and implement timely interventions, preventive measures, and treatment strategies upon definitive diagnosis.

IL‐8 gene polymorphisms (−251, +353, +781) were related to cancer susceptibility, especially −251 site and gastric cancer, suggesting these polymorphisms may offer value as biomarkers suitable for use when early detecting cancer. Besides, above significant polymorphisms of IL‐8 may have some potential clinical applications: such as some related inhibitors. Future studies may be able to apply these results to guide diagnostic and therapeutic approaches to abrogate cancer‐related risk.

Several limitations should be taken into consideration when interpreting the findings of the meta‐analysis. First, the modulation of cancer risk is influenced by the interactions among genes, gene–environment, and polymorphisms within the same gene. Therefore, future research endeavors should incorporate these factors to comprehensively understand cancer susceptibility. Second, it is imperative to incorporate various covariates such as sex, age, family history, environmental factors, cancer stage, and lifestyle into the analysis. Third, it should be noted that the control group consisted of individuals who did not strictly meet the criteria for being classified as healthy controls. Fourth, the investigation encompassed limited case–control studies regarding the polymorphisms (+678, +1633, +2767). Future research efforts should prioritize the examination of the above four polymorphisms. Fifth, case–control studies of small numbers of subjects, seeking to identify low‐penetrance susceptibility genes, may be confounded by interstudy variability and lack of reproducibility, so further work is required about larger patients combined with age‐ and sex‐matched controls to explore the trends and resolve apparent conflicts with other studies. Sixth, current analysis was the lack of haplotype reconstruction. Because, haplotypes are considered more powerful to detect susceptibility alleles than individual polymorphisms. Seventh, in some case–controls studies, the use of hospital controls is not ideal. The use of hospital controls probably has minimal effect on the allele frequencies, which may increase the potential bias. In final, further investigation is warranted to elucidate the underlying mechanisms, utilizing the available epigenetic data, related to the influence of distinct genotypes on tumor proliferation and invasion processes. In spite of these limitations, this meta‐analysis also possessed two notable advantages. First, in order to enhance the statistical power of the analysis, a substantial cohort of cases and controls was aggregated from multiple research investigations. Second, the inclusion of case–control studies in the present meta‐analysis was deemed satisfactory according to the predetermined selection criteria.

In conclusion, the present study evaluated the involvement of three polymorphisms (−251, +353, +781) of the IL‐8 gene in cancer risk, especially for gastric cancer. Hence, it is imperative to conduct additional meticulously planned and extensive investigations, specifically focusing on the interplay between genes and both genetic and environmental factors. Future investigations in this field are anticipated to yield enhanced and comprehensive insights into the correlation between genetic polymorphisms of the IL‐8 gene and susceptibility to cancer development.

## Author Contributions

**Bin Xun:** writing main manuscript; preparing figures. **Yidan Yan:** data curation (equal); formal analysis (equal). **Bin Xun:** validation (equal); visualization (equal). **Yidan Yan:** validation (equal); visualization (equal). **Bin Xun:** supervision (equal); writing – review and editing (equal).

## Ethics Statement

Approval of the research protocol by an Institutional Reviewer Board.

## Conflicts of Interest

The authors have stated explicitly that there are no conflicts of interest in connection with this article.

## Data Availability

All data and material in this study were available. In addition, the present work has not been published and not in consideration elsewhere. Also, the current work has been published as preprint https://www.researchsquare.com/article/rs‐3348999/v1.
